# Exposure to Zinc oxide nanoparticles during pregnancy induces oocyte DNA damage and affects ovarian reserve of mouse offspring

**DOI:** 10.18632/aging.101539

**Published:** 2018-08-28

**Authors:** Qiu-Yue Zhai, Wei Ge, Jun-Jie Wang, Xiao-Feng Sun, Jin-Mei Ma, Jing-Cai Liu, Yong Zhao, Yan-Zhong Feng, Paul W. Dyce, Massimo De Felici, Wei Shen

**Affiliations:** 1 College of Life Sciences, Institute of Reproductive Sciences, Qingdao Agricultural University, Qingdao 266109, China; 2 Animal Husbandry and Veterinary Station of Penglai City, Yantai 265600, China; 3 Institute of Animal Sciences, Heilongjiang Academy of Agricultural Sciences, HarbinHeilongjiang 150086, China; 4 Department of Animal Sciences, Auburn University, Auburn, AL 36849, USA; 5 Department of Biomedicine and Prevention, University of Rome ‘Tor Vergata’, Rome 00133, Italy; * Equal contribution

**Keywords:** Zinc oxide nanoparticles, oocyte, meiosis, DNA damage, ovarian reserve

## Abstract

Zinc oxide nanoparticles (nZnO) have been shown to have higher toxic effects likely due to their ion-shedding ability and low solubility under neutral conditions. In order to investigate whether exposure to nZnO during embryonic development affects ovary development, 12.5 day post coitum (dpc) fetal mouse ovaries were cultured in the presence of nZnO for 6 days. We found that the nanoparticles (NPs) accumulated within the oocyte cytoplasm in a dose dependent manner, caused DNA damage and apoptosis, and result in a significant decrease in oocyte numbers. No such effects were observed when the ovaries were incubated in the presence of ZnSO_4_ or bulk ZnO as controls. In addition, we injected intravenously 16 mg/kg body weight nZnO in 12.5 dpc pregnant mice on two consecutive days and analyzed the ovaries of fetuses or offspring at three critical periods of oogenesis: 17.5 dpc, 3 days post-partum (dpp) and 21 dpp. Evidence of increased DNA damage in pachytene oocytes in fetal ovaries and impaired primordial follicle assembly and folliculogenesis dynamics in the ovaries of the offspring were found. Our results indicate that certain types of NPs affect pre- and post-natal oogenesis *in vitro* and *in vivo*.

## Introduction

The widespread application of metal nanoparticles has greatly increased the chance of environmental exposure. Furthermore, NPs are found in everyday consumer products including paintings and electronic devises [1–4]. For this reason, potential toxicological effects of NPs have received increasing attention. It is believed that during embryo/fetal development is particularly vulnerable to the exposure of NPs due to the fact that they can penetrate the placental barrier and cause embryo toxicity [5]. The reproductive and developmental toxicity of carbon and silver NPs have been recently reviewed [6]. Meanwhile, the toxicity of nZnO has been investigated in various organs and tissues, including the testes in animal models [7–9]. Some of these studies demonstrated that nZnO may adversely impact the female reproductive system and fertility [10,11]. In this regard, we recently found that exposure of chick oocytes to nZnO inhibits their developmental capabilities following fertilization [12]. Little is known, however, of the consequences of maternal exposure to nZnO during the prenatal period and its effects on oogenesis in offspring.

Primordial germ cell (PGC) proliferation and fetal oocyte degeneration before and early after birth are regarded as important processes responsible for the establishment of a finite pool of primordial follicles. In mice, PGCs mitotically divide until 13.5 dpc at which time they reside in the fetal gonads and after which they immediately enter meiosis. Failure to properly form and repair meiotic DNA double-strand break (DSBs) result in gametes with alterations to chromosome structure or aneuploidy, which can lead to development disorders [13]. From 17.5 dpc to 4 dpp germ cell cysts undergo a process called “breakdown” which is associated with oocytes undergoing waves of programmed cell death (PCD). Therefore, oocytes with errors during chromosomal crossover might be eliminated by oocyte quality control mechanisms. Finally, another mechanism demonstrated to function as a developmental check point for oocytes, involves excessive retrotransposon activation (in particular of LINE-1) occurring as a consequence of the bulk demethylation taking place in PGCs before the beginning of meiosis [14]. As a consequence of PCD, only about one-third of germ cells produced following PGC/oogonia proliferation are present within the ovaries early after birth [15]. Alteration of meiosis and primordial follicle formation may increase the number of poor quality oocytes and lead to increased PCD, decreased ovarian reserve and pathologies such as premature ovarian inefficiency (POI) [16]. Although recent evidence indicates that postnatal neo-oogenesis is possible in some mammalian species [17], it is still largely accepted that the oocyte population forming the primordial follicles, during late pregnancy or early post birth, constitute a finite pool. It is therefore of great importance for mammalian females to efficiently establish the ovarian reserve during the pre- and early postnatal period and to maintain it for the rest of their reproductive lifespan.

In the present work, we studied whether maternal exposure to nZnO during embryo development affects oocyte DNA integrity and the establishment of the ovarian reserve in female offspring and using an *in vitro* ovary culture system investigated the possible underlying mechanisms.

## RESULTS

### nZnO penetrate into fetal ovaries and increase DNA damage and apoptosis in fetal oocytes

**Figure 1:**
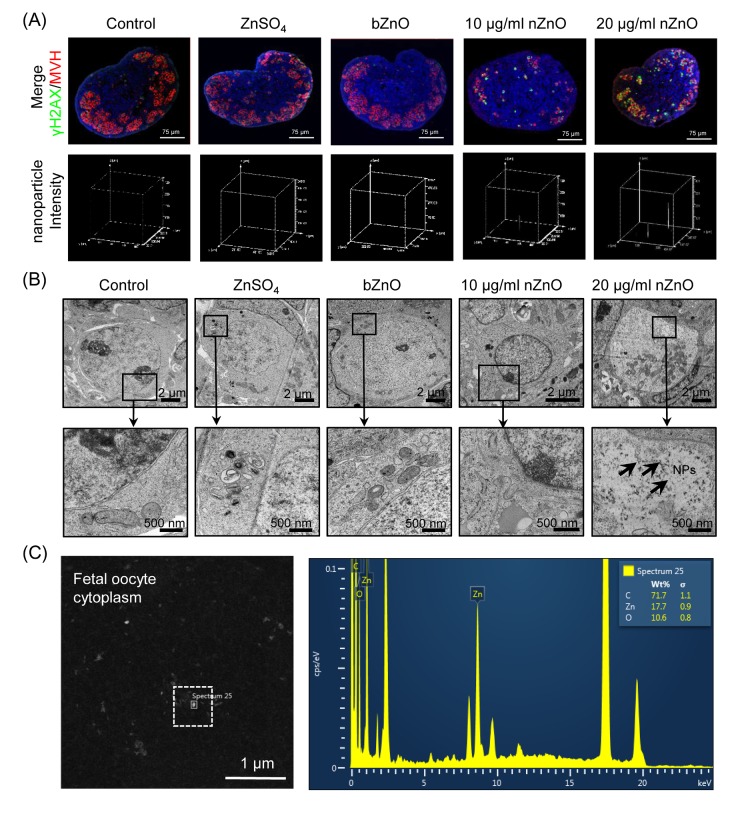
**Internalization of nZnO in fetal oocytes.** (**A**) Up: confocal reflection imaging of tissue sections of ovaries cultured for 6 days stained for MVH (red) and Hoechst 33342 (blue); nZnO was reflected and visualized as white dots. Down: 3-D plotting of nZnO intensity is presented with the z-axis indicating the intensity of nZnO within the ovary section and the interface of the x- and y-axes indicating the whole ovary section. (**B**) Ovary sections observed at TEM; NPs are not detectable in the control, ZnSO_4_ or bZnO whereas nZnO are recognizable as black particle in the oocyte cytoplasm (arrows). N: nucleus; C: cytoplasm. (**C**) Chemical characterization of nZnO nanoparticles with TEM equipped with energy dispersive spectrometer (EDX) within the oocyte cytoplasm.

To determine if nZnO could penetrate into the fetal mouse ovary we cultured 12.5 dpc ovaries *in vitro* in the presence of ZnSO_4_, bZnO or 10 and 20 µg/ml nZnO, for 6 days (ensure the molar weight of zinc is equal). Observations under an inverted microscope did not reveal any evident morphological alterations in ovaries incubated in the presence of nZnO, ZnSO_4_ or bZnO (Figure S2). Examination of ovary tissue sections immunostained for the oocyte specific protein MVH (a marker of germ cells) under a confocal reflection microscope, indicated that nZnO, but not ZnSO_4_ or bZnO, were accumulated in the ovaries in a dose independent manner (Figures 1A and Figure S3). In TEM sections, nZnO were observed in the oocyte cytoplasm (Figures 1B and Figure S4). The use of a TEM equipped with EDX, confirmed the presence of nZnO but not dye particles within the oocytes (Figure 1C). Although information about the endocytosis capabilities of fetal oocytes is lacking, this is likely to occur for nZnO since this is the usual route of internalization for similar NPs by cells [18].

**Figure 2:**
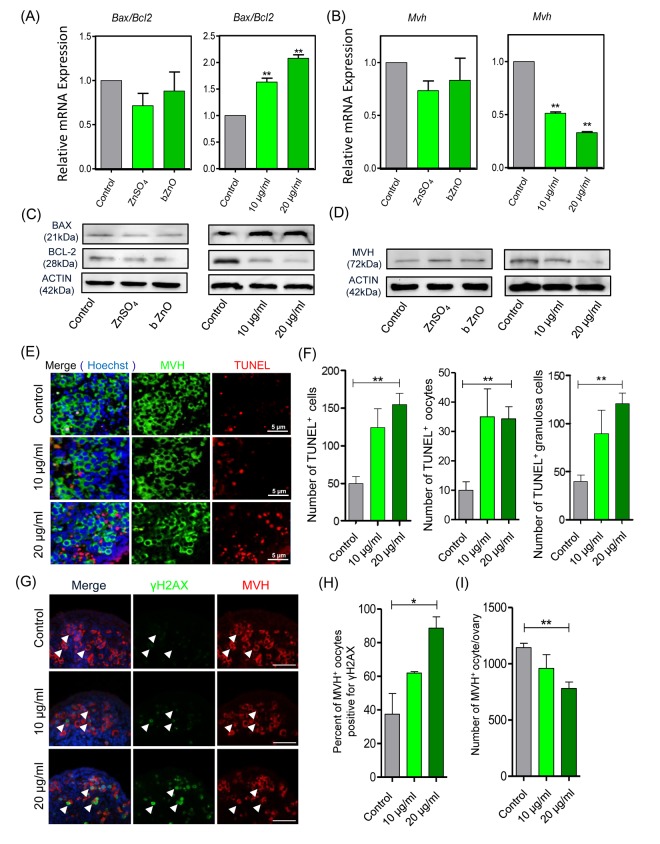
**Apoptotic and DNA damage markers in nZnO treated fetal oocytes *in vitro*.** (**A**-**D**) Representative q-RT-PCR and WB analyses of *Bax*, *Bcl-2* and *Mvh* of ovarian tissues cultured for 6 days; *Actin* or *Mvh* was used as housekeeping gene and loading control, respectively. (**E**) TUNEL-staining of the ovarian tissues after 6 days of culture with nZnO. (**F**) Number of TUNEL-positive total cells, TUNEL-positive oocytes and TUNEL-positive granulosa cells per section. (**G**) Co-immunostaining of DNA damage marker γH2AX (green) and germ cell marker MVH (red) in ovary sections after 6 day nZnO exposure, Hoechst 33342 (blue) was used for nuclei staining. (**H**) Percentage of MVH positive oocytes also stained for γH2AX. (**I**) Number of MVH positive oocytes/ovary.

The analysis of *Bax* and *Bcl-2* transcripts and their encoded proteins showed that their ratio were increased significantly in the ovaries cultured in the presence of nZnO, thus representing evidence of ongoing apoptosis. However, the ratio was not altered in the presence of ZnSO_4_ or bZnO (Figure 2A-2C). Moreover, the number of TUNEL-positive cells was significantly increased in ovaries incubated in the presence of 20 µg/ml nZnO in comparison to the untreated controls (Figure 2E and 2F). Double immunofluorescence on tissue sections of ovaries cultured for 6 days indicated that the number of MVH positive oocytes stained for γH2AX increased in the nZnO treated ovaries (Figure 2G and 2H). It is interesting that the amount of MVH protein decreased in the ovaries cultured in the presence of nZnO in a dose dependent manner, but not when ZnSO_4_ or bZnO were added to the culture medium (Figure 2D). Similarly, the number of MVH positive oocyte decreased in the ovaries cultured in presence of nZnO (Figure 2I).

### Exposure of nZnO increase DSBs in fetal oocytes both *in vitro* and *in vivo*

**Figure 3:**
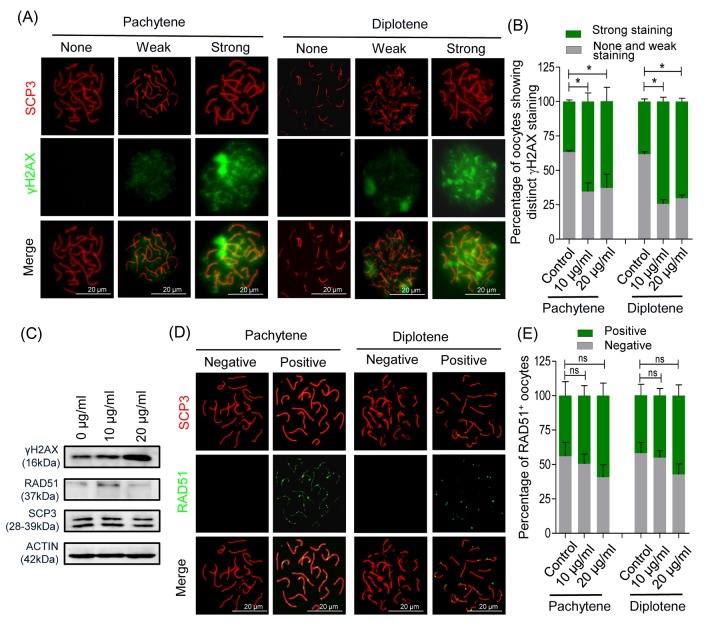
**nZnO induces DNA damage in fetal oocytes *in vitro*.** (**A**) Representative double IF of pachytene and diplotene oocytes for SCP3 (red) and γH2AX (green) obtained from ovarian tissues cultured for 6 days. (**B**) Quantification of oocytes showing distinct γH2AX staining. (**C**) Representative WB for the indicated proteins from ovarian tissues cultured for 6 days; actin was used as loading control. (**D**) Representative double IF of pachytene and diplotene oocytes for SCP3 (red) and RAD51 (green). (**E**) Quantification of oocytes showing RAD51 foci.

We confirmed that nZnO penetrate fetal ovaries and increase DNA damage and apoptosis of fetal mouse oocytes (Figure 2A-2I). We next analyzed cytospreads and confirmed a higher number of strongly positive γH2AX oocytes both at the pachytene and the diplotene stages in the ovaries exposed to nZnO (Figure 3A and 3B). Moreover, western blot (WB) analysis revealed that the overall γH2AX expression level increased in the whole ovaries (Figure 3C). Immunofluorescence for RAD51, an enzyme involved in homologous DNA recombination and repair [19], showed a slight but not significant increase in the number of oocytes showing fluorescent foci in the nZnO treated ovaries (Figure 3D and 3E); RAD51 protein amount in the whole ovaries showed similar results as revealed by WB analysis (Figure 3C). Despite these adverse effects of nZnO on the fetal ovaries and in particular on the oocytes, immunofluorescence analysis for SCP3 in cytospreads did not reveal alterations in the meiotic entry and progression within the exposed oocytes (Figure S5A and S5B). Likewise, qRT-PCR did not show significant variation in the mRNA level of genes encoding proteins involved in meiotic processes (Figure S5C).

**Figure 4:**
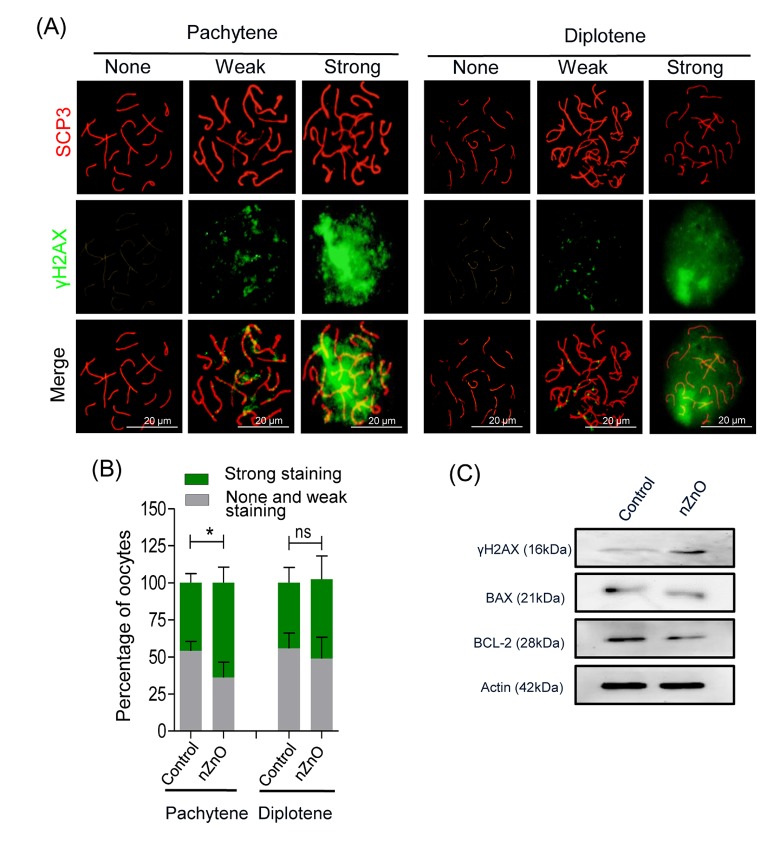
**nZnO expose increased DNA damage and BAX/BCL-2 ratio in fetal oocytes *in vivo*.** (**A**) Representative cytospreads of oocytes at the pachytene and the diplotene meiotic stage after nZnO intravenous injection of 12.5 dpc female mice. Chromosomes were stained for SCP3 (red) and DNA damage evidenced by γH2AX (green) staining. (**B**) Percentage of oocytes showing weak or strong γH2AX staining. (**C**) Representative images of western blotting analysis of BAX, BCL-2, and γH2AX. Actin was used as the loading control.

In order to investigate the effect of nZnO exposure on fetal oocytes *in vivo*, we intravenously injected 12.5 dpc pregnant mice with 16 mg/kg body weight nZnO on two consecutive days. At 17.5 dpc, fetuses were collected and ovaries dissected. The number and body weight of fetuses from mothers exposed to nZnO trended lower, but was not statistically significant (Figure S6A). Double immunofluorescent analysis of oocyte cytospreads with antibodies against the synaptonemal protein SCP3 and γH2AX, demonstrated that nZnO exposure significantly increased DSBs in oocytes at the pachytene, but not at the diplotene stage, in comparison to untreated controls (Figure 4A and 4B). Enhanced DNA damage in the whole ovaries exposed to nZnO was also suggested by the increased level of γH2AX expression evaluated by WB (Figure 4C). To sum up, exposure of nZnO increase DSBs in fetal oocytes both *in vitro* and *in vivo* (Figures 3, 4 and Figure S5D).

### The adverse effects on primordial follicle assembly and ovarian reserve after pregnant mothers exposed to nZnO

**Figure 5:**
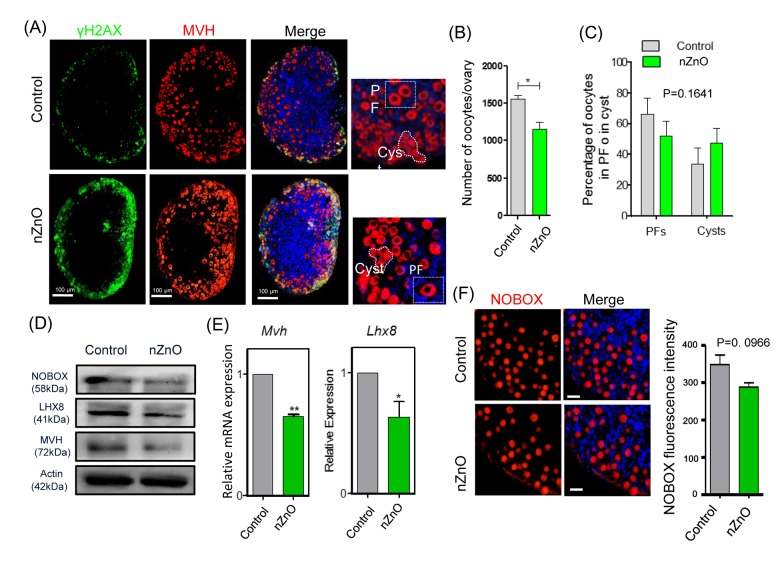
**nZnO expose decrease the oocyte number and affect oocyte specific gene expression** **in 3 dpp ovaries.** (**A**) Representative immunofluorescence pictures of MVH stained oocytes in sections of 3 dpp ovaries; Hoechst 33342 was used for nuclei staining; note oocytes within PF or in Cyst. (**B**) Number of oocytes per ovary. (**C**) Percentage of oocytes in PF or Cyst. (**D**) Representative WB of the indicated proteins in 3 dpp ovaries; (**E**) Quantitative RT‐PCR for *Mvh* and *Lhx8* mRNA levels in 3 dpp ovaries, *Actin* or *Mvh* was used as housekeeping gene and loading control. (**F**) IF for NOBOX (red) and fluorescence intensity of NOBOX in oocytes of control and nZnO exposed ovaries in 3 dpp ovaries; Hoechst 3342 (blue) was used for nuclei staining.

The immunofluorescent analysis of the 3 dpp ovaries of pups delivered by mothers exposed to nZnO, showed that the number of MVH positive oocytes was significantly decreased in comparison to untreated controls (Figure 5A-5C). Furthermore, the oocyte specific proteins MVH, NOBOX, and LHX8 and the transcripts *Mvh* and *Lhx8* in these ovaries also showed decreased expression (Figure 5D and 5E). NOBOX fluorescence intensity in single oocytes (Figure 5F), the mRNA expression of *Figα* and *Sohlh2*, and the body weight of fetuses from mothers exposed to nZnO trended lower, but were not significant different (Figures S6B and S6C). Other parameters such as DNA damage (double positive MVH and γH_2_AX oocytes), and BAX and BCL-2 protein expression in the whole ovaries were not altered by nZnO exposure (Figures S6D and S6E).

**Figure 6:**
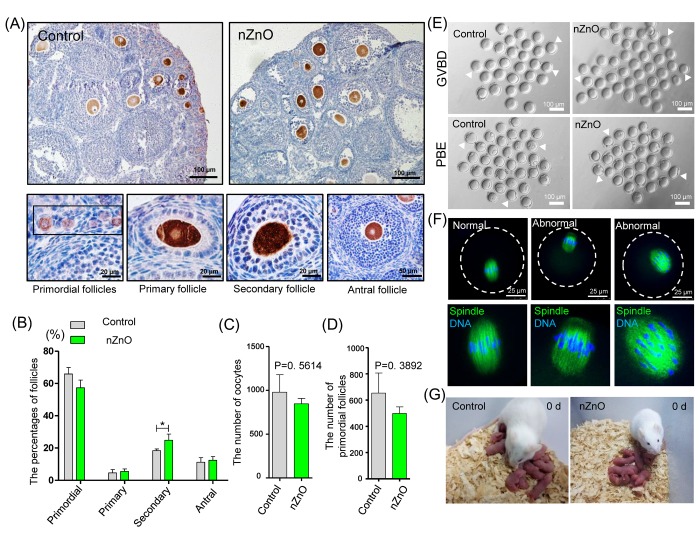
**nZnO expose alters follicle dynamics in 21 dpp ovaries.** (**A**) Identification of different type of follicles with immunohistochemistry of MVH positive oocytes in 21 dpp ovaries. (**B**) Percentage of different type of follicles in the same ovaries of (**A**). (**C**-**D**) Number of oocytes and PFs in the same ovaries of (**A**). (**E**) Representative images of GVBD and PBE. (**F**) Meiotic spindle morphologies in MI oocytes. (**G**) Representative morphologies of second filial generation.

We next investigated whether nZnO exposure altered the dynamics of the first folliculogenesis wave in the offspring ovaries. To this aim, we scored the number of oocytes present in primordial, primary, secondary and antral follicles in 21 dpp ovaries of the offspring of the nZnO exposed mothers (Figure 6A). In Figure 6B, it was shown that whereas no significant difference was detected in the percentage of primordial follicles, primary follicles and antral follicles, the percentage of secondary follicles was significantly higher in nZnO exposed ovaries than in the untreated controls (*P* < 0.05), suggesting accelerated follicle dynamics. Interestingly, in such ovaries, the total number of oocyte and primordial follicles was decreased although not significantly (Figure 6C and 6D).

Oocytes obtained from nZnO exposed ovaries of adult offspring (4 - 6 weeks), showed normal meiotic maturation capabilities evaluated by the rates of germinal vesicle breakdown (GVBD) and PBE (polar body exclusion) after 4 h and 12 h of *in vitro* culture (Figure 6E, Figure S6F and Figure S6G). Moreover, both in control and the nZnO exposed group, most oocytes showed normal spindle morphologies at the MI stage (Figure 6F and Figure S6H). Finally, no obvious differences in the F_2_ of control and nZnO exposed groups with regards to the number of fetuses and sex ratios were observed (Figure 6G and Figure S6I-S6J).

## DISCUSSION

**Figure 7:**
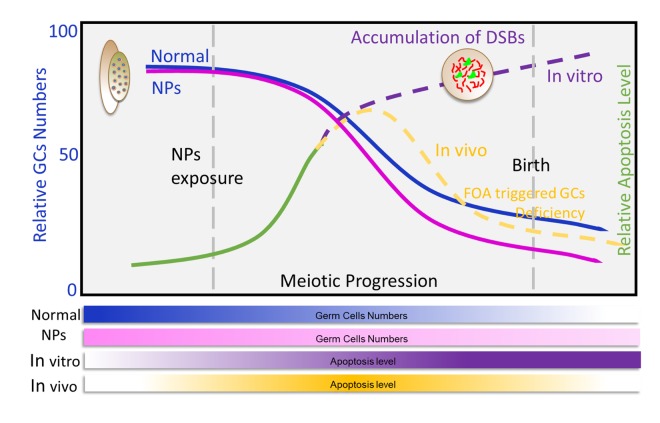
**Exposure to nZnOs during pregnancy induces the DNA damage of fetal oocytes and affects ovarian reserve of mouse offspring.** Maternal exposure to nZnO during embryo development affects the DNA damage of fetal oocytes and the establishment of the ovarian reserve in the female offspring using an *in vitro* ovary culture system or animal model.

Nanoparticles are widely used as antibiotic agents in textile, wound dressings, medical devices, and in appliances like refrigerators and washing machines [20]. They are heavily used due to their unique characteristics, such as high hydrophilicity, and ease of synthesis [21,22]. The increasing use of nanomaterials has raised concerns about the potential risks to human health, especially on the reproduction system [5,23,24]. Several studies have addressed the concerns of NPs exposure on reproductive health, however these studies focused on the impact of NPs on germ cell and pregnancy complications [5,20,25] (Figure 7). It is worth noting that fetuses are known to be more sensitive to environmental toxins than adults [26–28]. Furthermore, it has been suggested that chemicals can affect the maturation of germ cells, impair fertility, cause cancer, and disrupt the development of offspring [29,30]. The current study proved that nZnO exposure to pregnant mice perturbed fetal oogenesis and follicle development. We investigated several critical periods of oogenesis both *in vivo* and *in vitro* and it was found that exposure to nZnO could induce the accumulation of DSBs in fetal oocytes and decrease the ovarian follicle reservoir in fetal ovaries.

*In vitro* experiment results primarily showed that nZnO had toxicity on fetal oocytes. 12.5 dpc fetal ovaries were isolated and cultured with nZnO *in vitro*, and nZnO penetrated into the fetal ovaries and caused cytotoxicity to the germ cells. Consistent with previous studies [7,31–33], Exposure to nZnO mainly induced cellular DNA damage and as a consequence resulted in increased expression of apoptosis related proteins in the fetal ovaries. Since several types of cells exist within the fetal ovaries, we next investigated whether the nZnO penetrated and affected particular cell types. Noteworthy, the co-immunostaining analysis revealed that DNA damage signals mainly occurred in the germ cells, and TEM analysis also demonstrated that nZnO exposure caused obvious lesions in fetal oocytes. However, of great concern is that the orchestrated germ cell development is indispensable for successful establishment of the ovarian reservoir. The primordial follicle pool represents the lifelong reproductive reserve in the female ovary and *in vitro* exposure to nZnO affected fetal oocyte development. In particular, fetal ovary exposure to nZnO increased the accumulation of DSBs as shown by γH2AX expression during the first meiotic prophase (MPI). As a consequence of the cytotoxicity to germ cells it is reasonable to propose that germ cells with a high level of DNA damage underwent apoptosis leading to decreased germ cells in the fetal ovary.

To future confirm nZnO toxicity on pregnant mice, the germ cell number at 17.5 dpc (germ cells arrived in diplotene of MPI), 3 dpp (ovarian reservior establishment), 21 dpp (puberty), and 4 - 6 weeks (adult) [34,35] was compared. It was found that nZnO exposure increased the accumulation of DSBs in 17.5 dpc fetal oocytes. However, when the fetus developed to 3 dpp, the accumulation of DSBs in the oocytes was no longer detectable, and the percentage of primordial follicle (PF) was significantly decreased. The progression of follicular development was significantly altered in 21 dpp mice ovary in the nZnO exposured group with a lower percentage of PFs. In addition, the rate of GVBD, PBE and the percentage of spindle defects were examined. The developmental competence of offspring oocytes showed no difference between the nZnO exposured group and the untreated control group. Considering the fetal oocytes attrition (FOA), a conversed and physiologically normal event of fetal oogenesis [14], it may be proposed that nZnO induced accumulation of DSBs in fetal germ cells led to higher apoptosis. Therefore, the difference in apoptosis by 3 dpp was no longer detectable, while the nZnO exposed group showed a decreased ovarian reservoir. Notably, because of the FOA eliminate the “low-quality” oocytes (defective of DSBs caused by nZnO), thus, the number of oocytes surviving to birth are likely to normal oocytes and determine the fertility [36,37]. In a word, the continual deletion of inferior oocytes might guarantee the quality of oocytes and maximize the chances of reproductive success.

Although nZnO exposure induced oocyte damage under both *in vitro* and *in vivo* scenarios the underlying mechanisms differ: the number of germ cells were decreased due to apoptosis induced by the accumulation of DSBs in germ cells following nZnO exposure *in vitro*, while the elimination of the ovarian follicle reserve *in vivo* was mainly driven by the oocyte selection mechanisms.

Differing from males, females are born with a finite pool of ovarian follicles. Currently, more and more women are suffering from POI which is characterized by estrogen deficiency, menstrual irregularity, elevated serum gonadotropins, low serum estradiol and impaired fertility prior to 40 years of age [38,39]. Thus, the orchestrated establishment of the ovarian follicle reserve is of great importance for female reproductive lifespan and is known to be vulnerable to environmental toxins [40,41]. The accelerated elimination of the primordial follicle pool is of great concern, as exhaustion is irreversible and may cause POI [38,42]. Although the mechanisms underlying this phenomenon were not investigated in this study, we may propose that nZnO exposure during primordial follicle assembly alters the epigenetic pattern of oocytes, resulting in transgenerational epigenetic inheritance [43,44]. Similar studies have reported reproductive consequences to NPsexposure including for gold nanoparticles which significantly changed the global DNA methylation pattern in human embryonic stem cells [45]. However, the specific mechanisms require further study to elucidate the role that epigenetic modifications have during oocyte development.

In conclusion, the present results indicate that nZnO can affect pre- and post-natal oogenesis related processes and result in increased DNA damage and decrease the ovarian follicle reserve. The need remains, however, for investigation into the effects of different types of NPs on the development and functioning of the female gonad and of the possible consequences that exposure to such inorganic materials during pregnancy has on fertility in the offspring.

## MATERIALS AND METHODS

### Animals

CD1 mice were purchased from Beijing Vital River Laboratory Experimental Animal Technology Co. LTD (Beijing, China). Animal housing conditions were: 21 - 22 °C, 12 h dark /12 h light cycles, free access to water and food. 6-week female mice were mated overnight and the presence of the vaginal plug the next morning was considered as 0.5 dpc. All procedures involved in this study were approved by the Ethical Committee of Qingdao Agricultural University.

### Nanoparticles

nZnO were purchased from Beijing DK Nano Technology Co. LTD (Beijing, China), supplied as a white powder, it has a purity of 99.9% and a specific surface area 50 cm^2^ /g. bulk ZnO (bZnO; ~ 230 nm) particles were purchased from Beijing Sorlabio Life Science Co. LTD (YZ-111619, Beijing, China) and ZnSO_4_ was purchased from Sigma-Aldrich, Inc (Sigma, Z0251, USA). nZnO characterization was performed using transmission electron microscope (TEM, JEM-2100F, JEOL Inc., Japan) (Figure S1A) and scanning electron microscope (SEM, JSM-7500F, JEOL Inc.) (Figure S1B). The average nZnO diameter was ~ 30 nm whereas that of bZnO particles were ~ 230 nm (Figure S1C) as we previously described [46,47]. Before addition to the culture medium or animal injection, 4 mg nZnO was dissolved in 1 ml of α-Minimal Essential Medium (α-MEM, Hyclone, SH30265.01B, Beijing, China) and ultrasonicated for 15 min. 100 µl nZnO suspension was injected via tail vein on two consecutive days (12.5 dpc and 13.5 dpc). The dosage of nZnO was used according to previous studies [5].

### Ovary culture

Ovaries were isolated from 12.5 dpc mouse embryos and cultured according to previously described procedures [48]. The culture medium was α-MEM, supplemented with 10% fetal bovine serum (FBS, Gibco, 10099-141, USA), 0.23 mM sodium pyruvate (Hyclone, SH40003-12) and 100 IU/ml of penicillin and 100 mg/ml of streptomycin sulfate, 10 mIU/ml follicle stimulating hormone (FSH; RD, 5925-FS, MN, USA). Half ovaries were cultured in a 24 well plate for six days in a humidified incubator at 37 °C, 5% CO_2_ in air and medium changed every other day.

### Oocyte culture

Germinal vesicle (GV)-intact oocytes were isolated from 4 - 6 week female mice in M2 medium (Sigma, M7167) containing 1 µM milrinone (Amyjet, USA) to prevent germinal vesicle breakdown (GVBD),after washing three times with M2 medium (Macgene, CE003, Beijing, China), oocytes were cultured at 37 °C, 5% CO_2_ atmosphere. After 8.5 h, oocytes were fixed in 4% paraformaldehyde for 30 min, and permeabilized in phosphate buffer saline (PBS) supplemented with 0.1% Triton X-100 for 20 min. Oocytes were then incubated in PBS containing 1% bovine serum albumin (BSA, Solarbio, A8020, Beijing, China) overnight at 4 °C. After three final washings, they were incubated with FITC anti-α-tubulin antibody (Santa Cruz, USA) for 2 h at room temperature and 10 min in Hoechst 33342 (Beyotime, C1022, Nantong, China) in the same buffer and finally observed under an Olympus fluorescence microscope (BX51, Tokyo, Japan).

### Oocyte cytospreads

Fetal oocyte cytospreads were performed as previously described [49]. Slides were incubated with the first antibody against rabbit/mouse SCP3 (rabbit polyclonal, Novus Biologicals Littleton, NB300-232, USA; or mouse polyclonal, Abcam, ab97672, USA), γH2AX (mouse polyclonal, Abcam, ab26350) or RAD51 (rabbit polyclonal, Abcam, ab133534), for 8 h at 37 °C. After overnight blocking with TBS supplemented with 10% goat serum (Boster, AR009, Wuhan, China), Cy3-labeled goat anti-rabbit (Beyotime, A0516) were used to label SCP3/RAD51 and FITC-conjugated goat anti-mouse antibodies (Beyotime, A0568), were used to label γH2AX/SCP3 at 1:100 dilution for 30 min at 37 °C in the dark; DNA was stained with Hoechst 33342. Pictures were taken with an Olympus fluorescence microscope (BX51).

### Ovary immunostaining

For immunostaining, ovaries were fixed with 4% paraformaldehyde, processed following standard paraffin embedding and serially sectioned at 5 μm. Slides were deparaffinized in xylene, rehydrated in a gradient ethanol series before antigen retrieval with 0.01 M sodium citrate buffer at 96 °C for 10 min. For immunofluorescence (IF), after blocking procedure, the slides were incubated with first antibody against mouse MVH (rabbit polyclonal, Abcam, ab13840) and γH2AX (Abcam, ab26350) overnight at 4 °C; CY3-labeled goat anti-rabbit (Beyotime, A0516) or FITC-conjugated goat anti-mouse secondary antibody (Beyotime, A0568), were used in the next morning at a dilution of 1:200; Hoechst 33342 was used for nuclei staining. Oocytes in cysts or into primordial follicles were scored in five section as previous described [50,51]. Images were obtained using an Olympus fluorescence microscope (BX51) or a Leica Laser Scanning Confocal Microscope (Leica TCS SP5 II, Wetzlar, Germany). Reflection analyses of ovary tissue sections were performed according to a previously described procedure [52,53]; the signal intensity of nZnOs were compared using 3D intensity analysis tools provided by Leica Laser Scanning Confocal Microscope Software [54,55].

For immunohistochemistry (IHC), after blocking, sections were exposed to 3% H_2_O_2_ for 10 min, blocked with BDT (3% BSA, 10% normal goat serum in TBS) for 30 min and incubated with first antibody against rabbit MVH (Abcam, ab13840) at 4 °C overnight. After careful washing, horseradish peroxidase (HRP)-conjugated goat anti-Rabbit IgG (Beyotime, A0258) was added at room temperature for 45 min. DAB (ZSGB-BIO, Beijing, China) was added to the slides and incubated for 5 - 10 min at room temperature. Hematoxylin was used for nuclei staining and the slides mounted with neutral balsam (Sinopharm, Shanghai, China).

### TUNEL assay

TUNEL staining was performed using "Bright Red Apoptosis Detect Kit" (Vazyme, A113-02, China). Briefly, ovary sections were heated at the 60 °C in an air oven for 2 h, washed in xylene and rehydrated through a series of ethanol and double distilled water. The samples were then treated with proteinase K for 15 min at room temperature, rinsed twice with PBS and incubated for 60 min at 37 °C in the dark in 100 µl of the TUNEL reaction mixture; nuclei were counterstained with Hoechst 33342.

### TEM and SEM

For transmission electron microscope (TEM) analysis, ovary tissues were fixed with 2% glutaraldehyde in sodium phosphate buffer (pH 7.2) for 2 h at 4 °C and then processed following standard procedures. Samples were sectioned with a Leica ultramicrotome (Leica EM UC7, Wetzlar, Germany) at 50 nm, transferred to a copper grids and stained with uranyl acetate. Pictures were taken with a HITACHI HT7700 transmission electron microscope (HITACHI, Tokyo, Japan) at an accelerating voltage of 80 kV.

For scanning electron microscope (SEM) analysis, samples were mounted on a SEM-specific stubs and samples coated with platinum with an auto fine coater (JEOL). Pictures were taken using a SEM (JEOL) equipped with an EDX detector (Oxford Instruments, Oxford, UK).

### Western blotting

Western blotting (WB) was as previously described [56]. Briefly, ovaries were collected and dissociated with RIPA lysis solution (Beyotime, P0013), SDS-PAGE was performed with a 4% stacking gel and a 10% separating gel for 120 min at 100 V. Proteins were then electrophoretically transferred onto polyvinylidene fluoride membrane and blocked with TBST supplemented with 5% BSA, after blocking, first antibody against BAX (Cell Signaling, 2772S, Beverly, USA), BCL-2 (Beyotime, AB112), NOBOX (Abcam, ab41521), LHX8 (Sigma, SAB2101342), MVH and ACTIN (Sangon, D110001, Shanghai, China) were incubated at 4 °C overnight. HRP-conjugated goat anti-rabbit (Beyotime, A0258) or anti-mouse (Beyotime, A0216) were then incubated at 1:2000 dilution in TBST in the next day at room temperature. Chemiluminescence of target proteins was performed with the enhanced chemiluminescence (ECL) detection system (ProteinSimple, San Jose, CA, USA).

### Quantitative real-time PCR (qRT-PCR)

Total RNA was extracted from ovarian tissues using RNA prep pure Micro Kit (Aidlab, RN07, Beijing, China), according to the manufacturer’s instructions. Reverse transcription was performed using TransScript^®^ One-Step gDNA Removal Kit and cDNA Synthesis Kit (TransGen, AT311-03, Beijing, China) following the manufacturer’s instructions. All primers used in this were listed in Table S1 and qRT-PCR were performed with LightCycler 480 II (Roche, LC480) using the LightCycler^®^ 480 SYBR Green I Master Kit (Roche, 04887352001). Reaction condition of q-PCR was set at 95 °C for 10 min, followed by 55 cycles at 95 °C for 10 s, 60 °C for 30 s, and finally cooling at 4 °C. Relative gene expression was calculated using the 2^-△CT^ method and normalized to *β-actin* or *Mvh*, each sample contained three technical repeats [57].

### Statistical analysis

All statistical analysis data involved in this study were performed in triplicate and variance and statistical comparisons were determined with GraphPad Prism 5.0 software (GraphPad Software, San Diego, CA, USA). Student’s two-tailed *t*-test was used to determine statistical significance between means and a significant difference was considered at *P* < 0.05 for all tests.

### Availability of data and materials

The datasets used and/or analyzed during the current study are available from the corresponding author on reasonable request.

## Supplementary Material

Supplementary Figures

Supplementary Table
