# Supplementary material for: Exposure to Zinc oxide nanoparticles during pregnancy induces oocyte DNA damage and affects ovarian reserve of mouse offspring
Source: Aging (Albany NY). 2018 Aug 28;10(8):2170–89. doi: 10.18632/aging.101539 (PMC6128443; doi:10.18632/aging.101539)
Supplement: Supplementary Figures [file aging-10-101539-s001.pdf]

SUPPLEMENTARY FIGURES

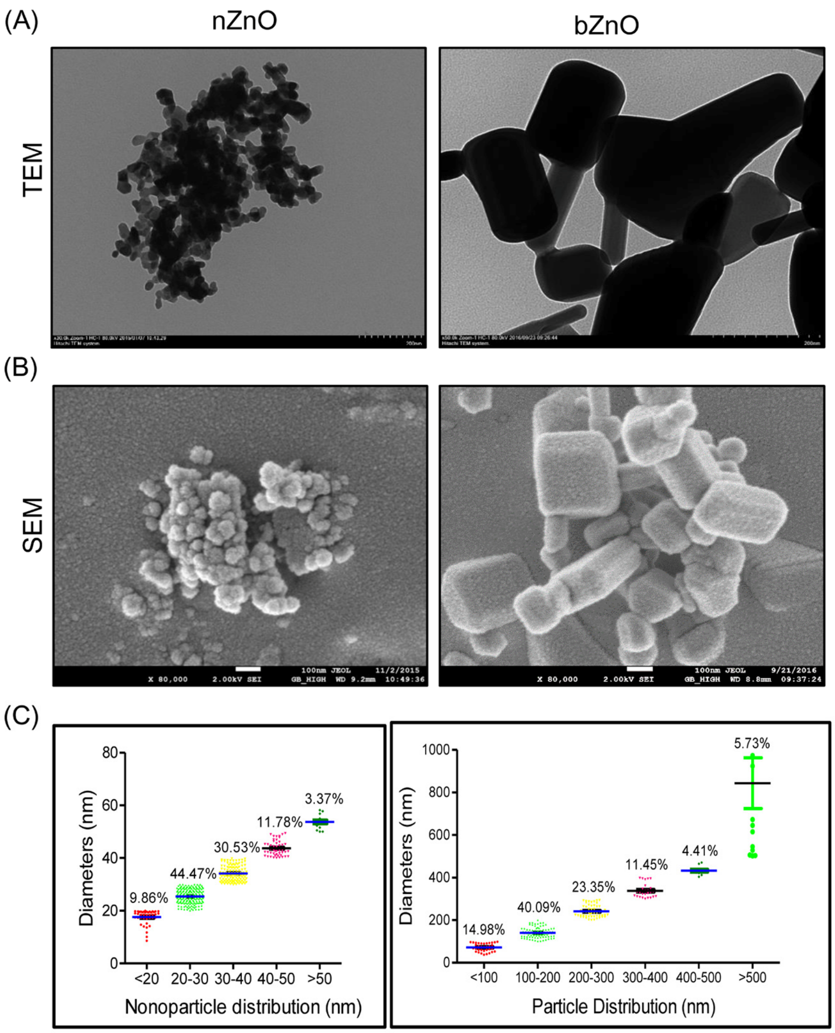

Figure S1. Characterization of nZnO and bZnO particles. (**A**) TEM analysis of nZnO and bZnO. (**B**) SEM analysis of nZnO and bZnO. (**C**) Size distribution of nZnO and bZnO.

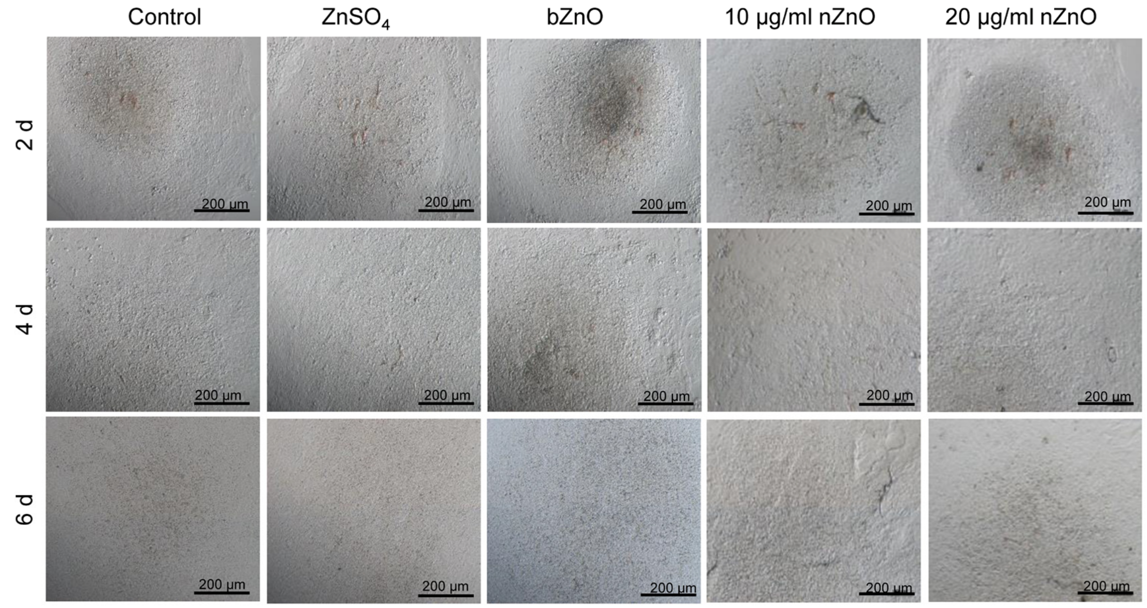

Figure S2. Morphology of control and ZnSO4, bZnO and nZnO exposed 12.5 dpc ovaries after 2, 4 and 6 days of culture.

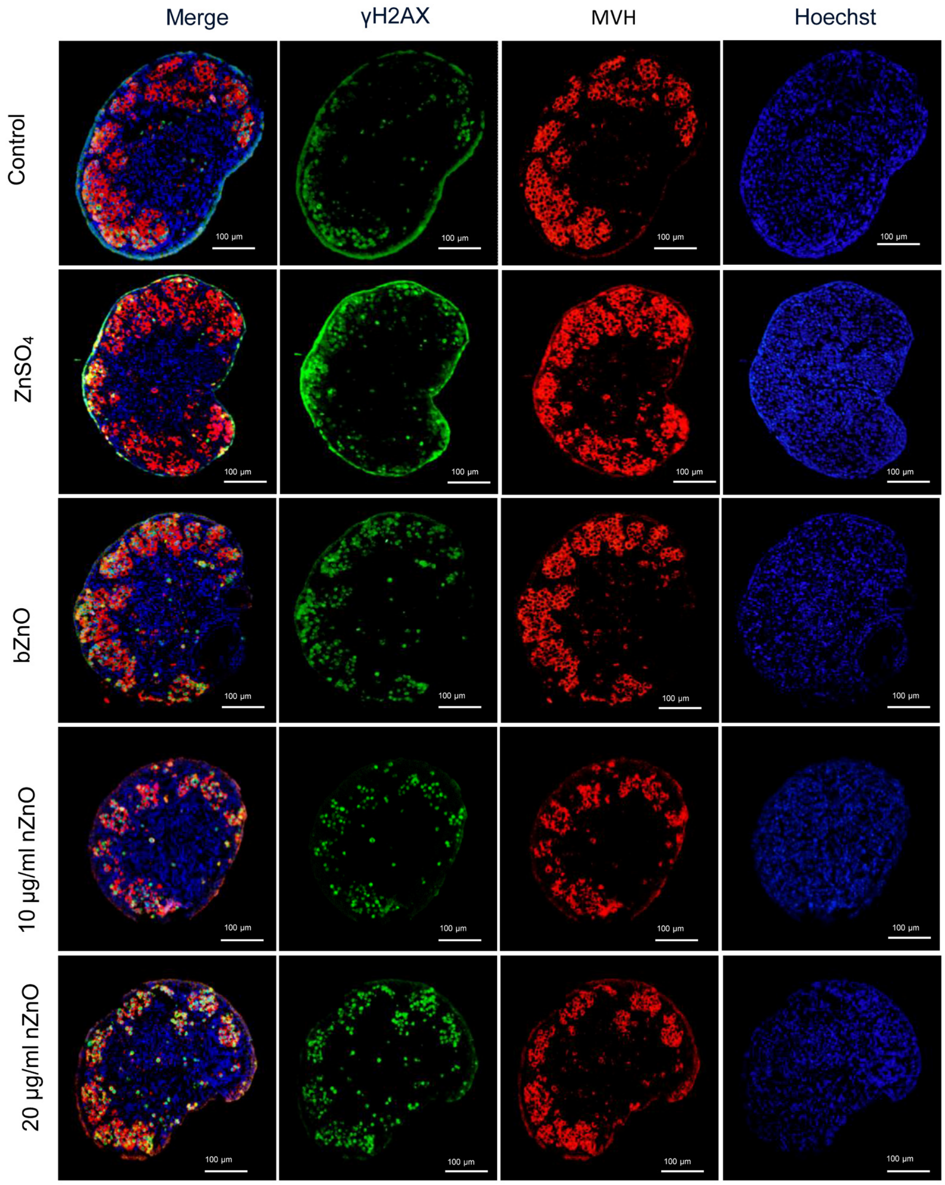

Figure S3. Ovaries cultured for 6 days stained for MVH (red) and γH2AX (green).

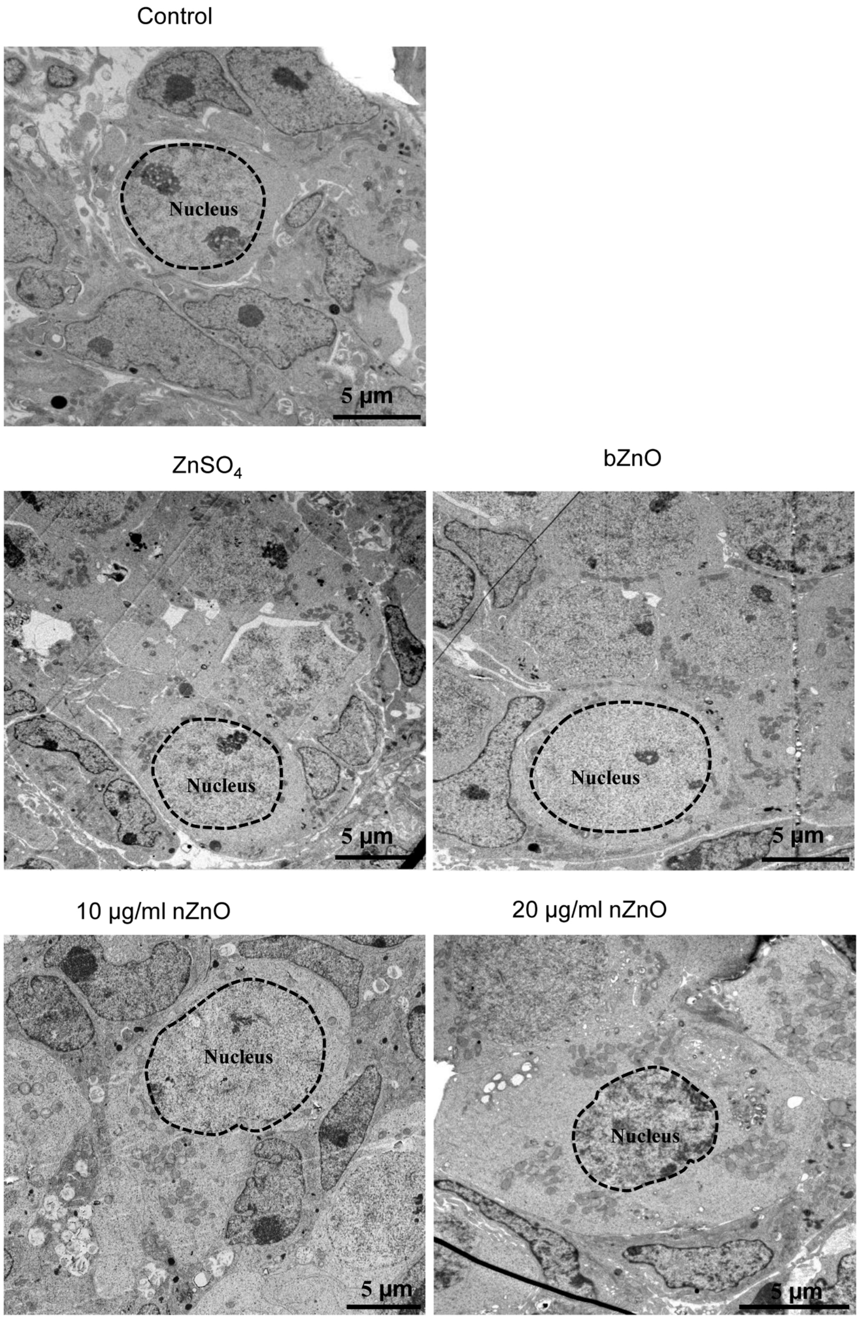

Figure S4. Ovary sections observed at TEM (dotted circle: nucleus).

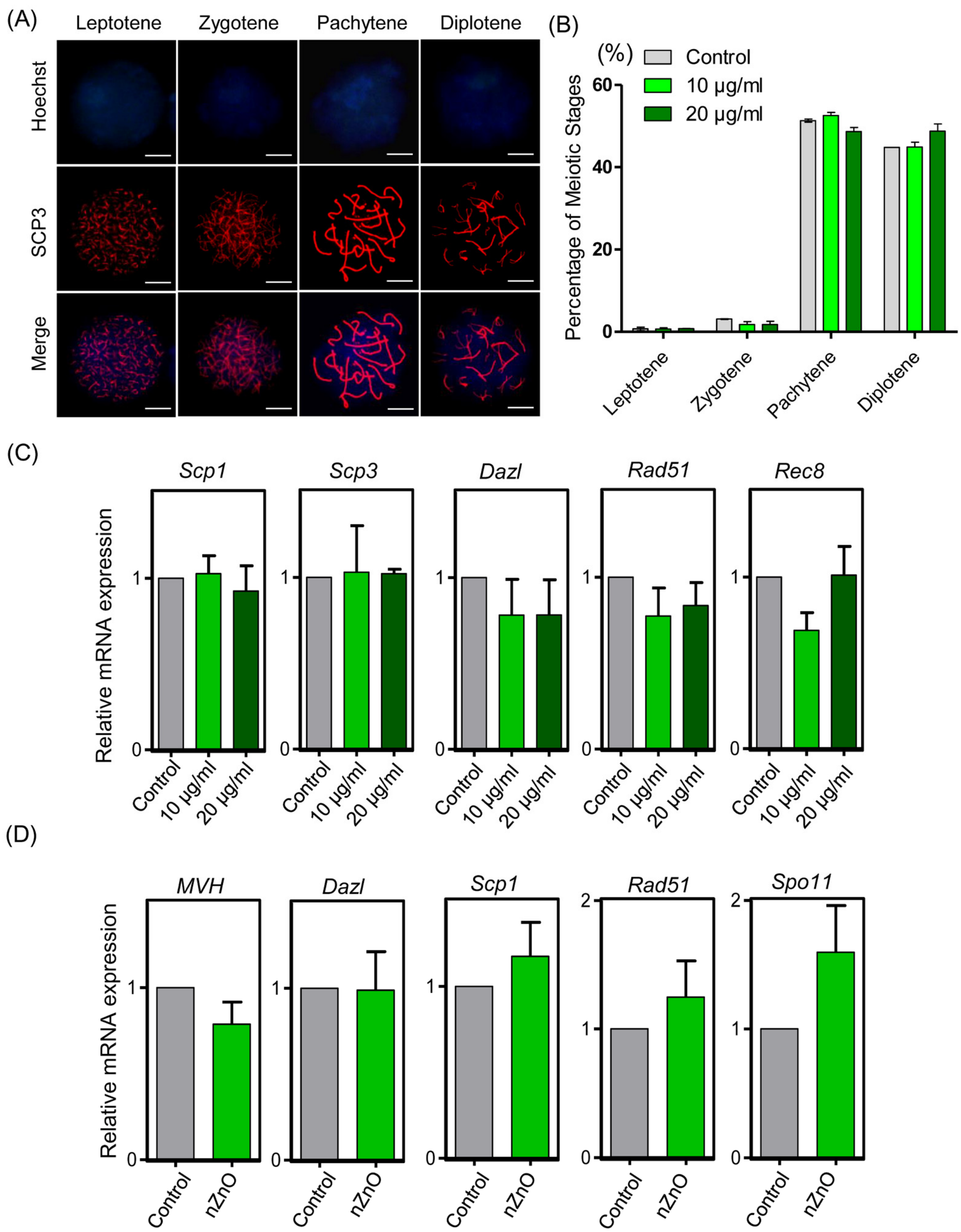

Figure S5. (**A**) Representative morphologies of leptotene, zygotene, pachytene, and diplotene oocytes. Chromosomes were stained with SCP3 （red） and DNA with Hoechst 33342 (blue). (**B**) Percentage of oocytes at different stages of meiosis obtained from ovaries cultured for 6 days as reported in the text. (**C**) Representative qRT-PCR of the indicated genes in ovarian tissues after 6 days of culture; *Actin* or *Mvh* was used as housekeeping gene. (**D**) qRT-PCR mRNA of genes encoding proteins typical of meiotic oocytes in 17.5 dpc fetal ovary. Expression was calculated relatively to the expression of *Actin* or *Mvh* as housekeeping gene in at least three independent experiments.

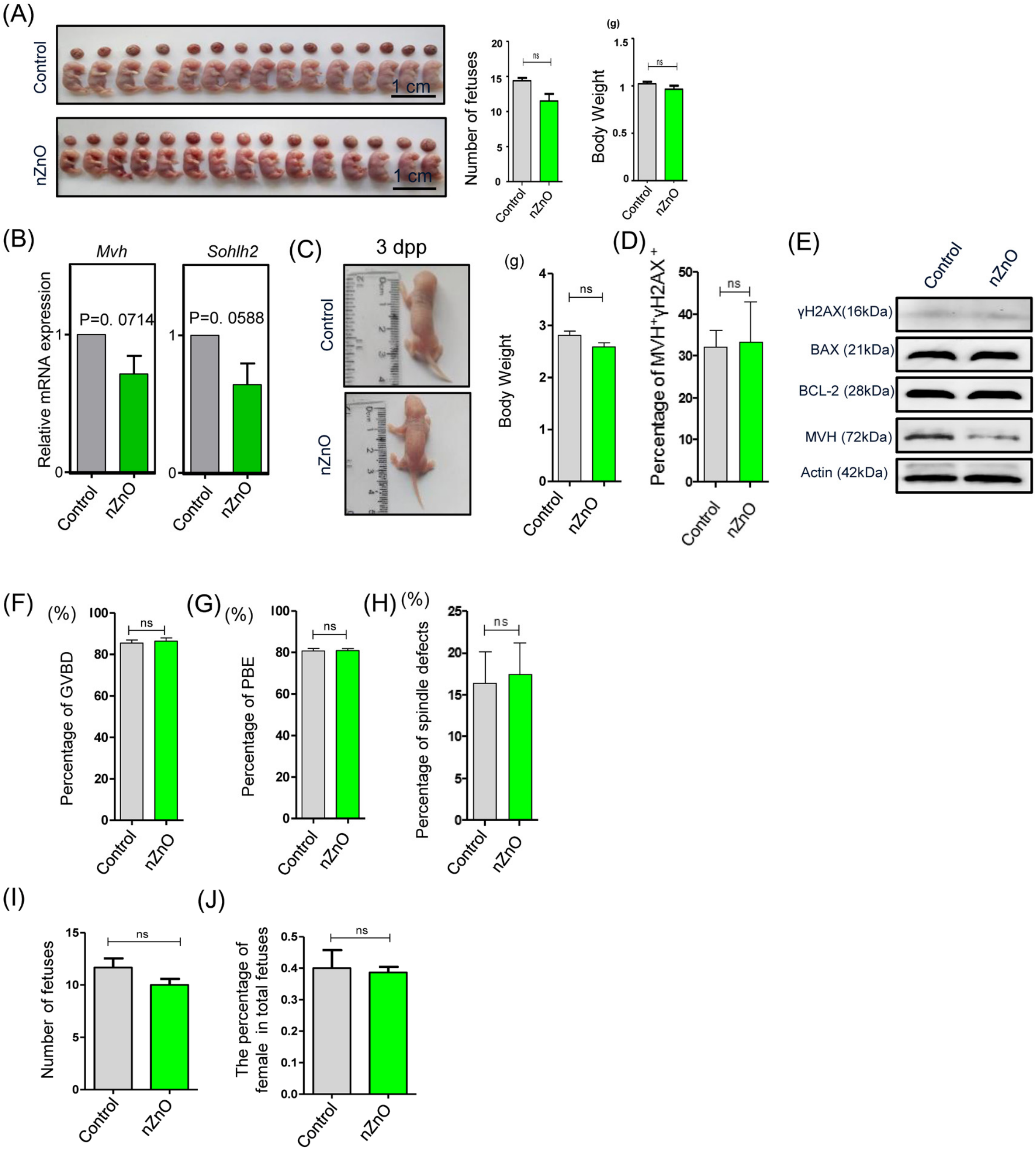

Figure S5. (**A**) Representative morphologies of leptotene, zygotene, pachytene, and diplotene oocytes. Chromosomes were stained with SCP3 （red） and DNA with Hoechst 33342 (blue). (**B**) Percentage of oocytes at different stages of meiosis obtained from ovaries cultured for 6 days as reported in the text. (**C**) Representative qRT-PCR of the indicated genes in ovarian tissues after 6 days of culture; *Actin* or *Mvh* was used as housekeeping gene. (**D**) qRT-PCR mRNA of genes encoding proteins typical of meiotic oocytes in 17.5 dpc fetal ovary. Expression was calculated relatively to the expression of *Actin* or *Mvh* as housekeeping gene in at least three independent experiments.
