# Supplementary material for: Exposure to Zinc oxide nanoparticles during pregnancy induces oocyte DNA damage and affects ovarian reserve of mouse offspring
Source: Aging (Albany NY). 2018 Aug 28;10(8):2170–89. doi: 10.18632/aging.101539 (PMC6128443; doi:10.18632/aging.101539)
Supplement: Supplementary Table [file aging-10-101539-s002.pdf]

SUPPLEMENTARY TABLE

Table S1. Primers for quantitative real-time PCR.

Genes	Forward primer	Reverse primer	Genebank Accession number
β-actin	TCGTGGGCCGCCCTAGGCAC	TGGCCTTAGGGTTCAGGGGGG	NM_007393.5
Mvh	TCAGACGCTCAACAGGATGT	ACTGGATTGGGAGCTTGTGA	NM_006517524.2
Scp1	GCGAAGATTGCTTTGGAGAC	GCAGATGCCCGCAGATTAT	NM_011516.2
Scp3	GGGGCCGGACTGTATTTACT	AGGCTGATCAACCAAAGGTG	NM_011517.2
Rad51	ACCAGACCCAGCTCCTTTAC	CAAGTCGAAGCAGCATCCTC	NM_011234.4
Spo11	TACTGCTGTGCCGACTAACA	GTAGGGATCTGCATCGACCA	NM_001305434.1
Rec8	TGATATGGAGGAGGCTGACC	GCAGCCTCTAAAAGGTGTCG	NM_020002.3
Dazl	ATCAGCAACCACAAGTCAAGG	GAGACAAATCCATAGCCCTTCG	NM_010021.5
Bax	ATGCGTCCAAGGAAGACTGAG	CCCCAGTTGAAGTTGCCATCAG	NM_007527
Bcl-2	GCAGAGATGTCCAGTCAG	CACCGAACTCAAAGAAGG	NM_009741.5
Lhx8	CAGTTCGCTCAGGACAACAA	CCTGCAGTTCTGAAACCACA	NC_000069.5
Fig-α	ACAGAGCAGGAAGCCCAGTA	TGGGTAGCATTTCCCAAGAG	NM_012013.1
Sohlh2	TCTCAGCCACATCACAGAGG	GGGGACGCGAGTCTTATACA	NM_028937.3
